# Variations of Bacterial Community Composition and Functions in an Estuary Reservoir during Spring and Summer Alternation

**DOI:** 10.3390/toxins10080315

**Published:** 2018-08-06

**Authors:** Zheng Xu, Shu Harn Te, Cong Xu, Yiliang He, Karina Yew-Hoong Gin

**Affiliations:** 1 School of Environmental Science and Engineering, Shanghai Jiao Tong University, Shanghai 200240, China; xuzheng-2004@163.com (Z.X.); xucong90@sjtu.edu.cn (C.X.); 2 NUS Environmental Research Institute (NERI), National University of Singapore, Singapore 138602, Singapore; eritsh@nus.edu.sg (S.H.T.); ceeginyh@nus.edu.sg (K.Y.-H.G.); 3 Department of Civil and Environmental Engineering, National University of Singapore, Singapore 138602, Singapore

**Keywords:** reservoir, Yangtze estuary, 16S rRNA gene sequencing, shotgun metagenomic sequencing, bacterial community, microbial metabolisms

## Abstract

In this study, we focused on the dynamics of bacterial community composition in a large reservoir in the Yangtze estuary during spring and summer seasons, especially the variations of functional mechanisms of microbial community during the seasonal alternation between spring and summer. Both 16S rRNA gene sequencing and shotgun metagenomic sequencing technology were used for these purposes. The results indicated that obvious variations of bacterial community structures were found at different sites. Particle-associated bacterial taxa exhibited higher abundance at the inlet site, which was closer to the Yangtze River with a high level of turbidity. In other sites, *Synechococcus*, as the most dominant cyanobacterial species, revealed high abundance driven by increased temperature. Moreover, some heterotrophic bacterial taxa revealed high abundance following the increased *Synechococcus* in summer, which indicated potential correlations about carbon source utilization between these microorganisms. In addition, the shotgun metagenomic data indicated during the period of seasonal alternation between spring and summer, the carbohydrate transport and metabolism, energy production and conversion, translation/ribosomal biogenesis, and cell wall/membrane/envelope biogenesis were significantly enhanced at the exit site. However, the course of cell cycle control/division was more active at the internal site.

## 1. Introduction

Estuary reservoirs, as important water sources for estuarine cities, are strongly influenced both by terrestrial and coastal environmental changes [1,2,3,4,5]. In the estuarine area, large accounts of organic matter originate (including N/P nutrients) from land and rivers, flowing through these systems and, finally, into oceans [2,6,7]. In addition, during some special seasons, the salt water invaded into the estuary because of the declined water levels of the river, which resulted in a high level of concentrations of salt ions in these areas [5,8]. Due to the unique geographical locations, the microbial community compositions within estuary reservoirs are very different from microbial community structures within lakes and oceans [9,10].

In estuary ecosystems, bacterial community plays important role in the microbial food web, such as recycling and consuming organic matters [9,11]. Research has indicated that the distinctly different distributions of particle-associated bacteria and free-living bacterial community in estuary areas, have been strongly affected by environmental factors such as turbidity and organic matters [11,12,13]. Although the microbial community composition in estuary aquatic ecosystems was widely studied and have got certain achievements in recent years, there is still a larger number of unclassified bacterial taxa and unknown ecological functions in estuary systems compared with terrestrial, inland lake, and ocean studies [14,15,16,17].

In Addition, Cyanobacteria as one of the most dominant members within the bacterial community should be paid more attention to in aquatic ecosystems, which could possible to form harmful cyanobacterial blooms when the environmental conditions became suitable in water bodies. Although the harmful cyanobacterial species (such as *Microcystis* and *Anabeana*) in freshwater lakes have been widely studied, the *Synechococcus* as one of the most dominant cyanobacterial species in estuarine and marine environments has been less studied. Especially, some strains of *Synechococcus* has been found to have toxicity effects on other marine organisms in recent years [18,19,20]. Related studies indicated that during the period of cyanobacterial proliferation, obvious variations of bacterial community composition were found in water bodies [21,22]. This implied that the functional mechanisms and ecological roles of the bacterial community changed in the process of cyanobacterial proliferation, which might correlate with nutrient utilization and spatial competition.

In this study, we utilized systematic methods that including high-throughput sequencing, molecular ecological network and metagenomics to reveal the composing characteristics of the bacterial community in the estuarine reservoir and identify the categories of dominate members within these complex bacterial communities in temporal and spatial scales. Additionally, we evaluated the effects of water environmental factors on bacterial community composition. Moreover, we explored the variations of functional metabolic mechanisms within the microbial community from later spring to early summer, which was the period of cyanobacterial proliferation.

## 2. Results

### 2.1. Physico-Chemical Parameters and Environmental Factor in QCS Reservoir

**Figure 1:**
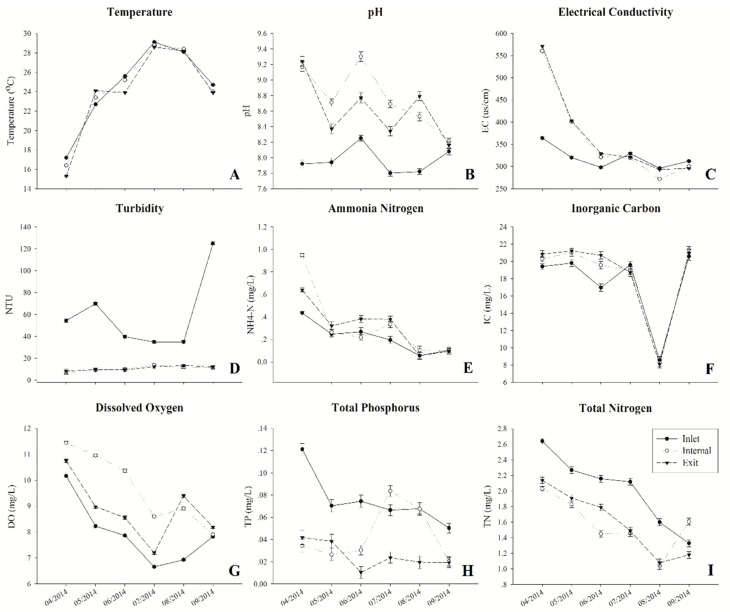
Water chemistry and environmental parameters. (**A**) Temperature, (**B**) pH, (**C**) electrical conductivity (EC), (**D**) turbidity (NTU), (**E**) ammonia nitrogen (NH_4_-N^+^), (**F**) inorganic carbon (IC), (**G**) dissolved oxygen (DO), (**H**) total phosphorus (TP), and (**I**) total nitrogen (TN).

During the sampling period, water temperature varied from 15.3 to 29.1 °C, which increased rapidly from April to July and decreased gradually from July to September at all three sites (Figure 1A). Fluctuating pH changes were both found at all sites, which ranged from 7.8 to 9.3. In addition, the pH at both internal and exit sites were much higher than at the inlet site (Figure 1B). Although the electrical conductivity (EC) exhibited obviously decreased trends at all three sampling sites from April to September, the value of EC at the inlet site was much lower than other sites during spring (April–June) (Figure 1C). The turbidity both at internal and exit sites were relatively stable during the whole sampling period, which ranged from 6.83 NTU to 13.7 NTU. In contrast, the turbidity at the inlet site (34.9–125 NTU) was obviously higher than the other sites and remarkably increased from August to September (Figure 1D). The concentrations of ammonium nitrogen (NH^+^_4_-N), inorganic carbon (IC), dissolved oxygen (DO), and total nitrogen (TN) decreased obviously when water temperature increased (Figure 1E–H). Among these environmental factors, higher levels of NH^+^_4_-N, IC and DO were observed both at internal and exit sites. Especially, the concentration of DO was obviously higher at the internal site from April to July. In addition, both concentrations of TN and total phosphorus (TP) obviously decreased from the inlet to other sites, with the exception of TP concentration in July and August at the internal site (Figure 1H–I). 

### 2.2. The Variations of Chlorophyll-α Concentrations in the QCS Reservoir

**Figure 2:**
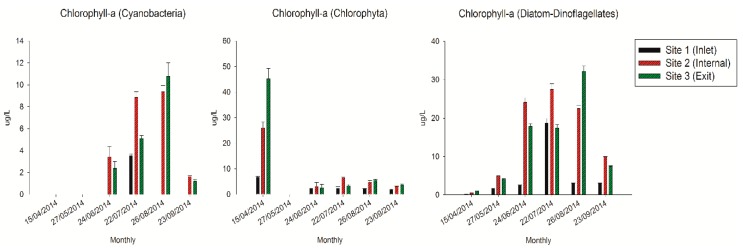
The concentrations of chlorophyll-α in the QCS Reservoir.

In this study, the concentrations of chlorophyll-α from different algae exhibited distinct variation tendencies inside the reservoir (Figure 2). The cyanobacterial chlorophyll-α exhibited relative higher concentrations during July and August compared with other periods, and reached the maximum value at 10.8 µg/L at the exit site in August. While the chlorophyll-α of Chlorophyta only appeared higher concentration in April at the exit site (44.8 µg/L). In contrast, the chlorophyll-α of diatoms and dinoflagellates exhibited obviously higher concentrations from June to August, especially at the internal and exit sites with an average value of 25.1 µg/L.

### 2.3. Dynamic Analysis of Bacterial Community Composition based on the 16S rRNA Sequencing Data

**Figure 3:**
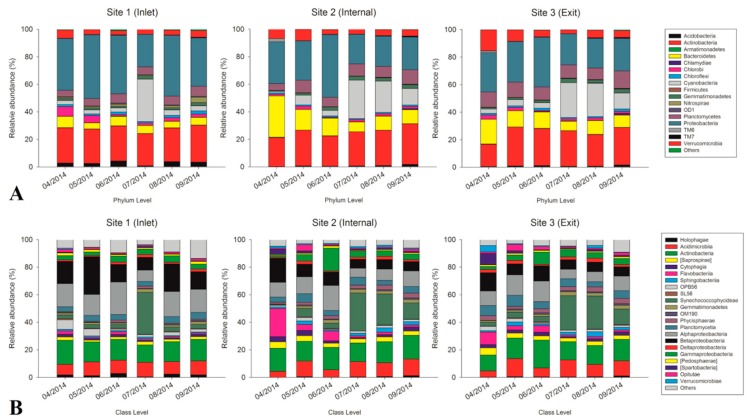
(**A**,**B**) Relative abundance of 16S rRNA bacterial OTUs across the whole sampling period ((**A**). phylum level, (**B**). class level).

Based on bacterial community composition analysis assessed by sequencing of V4 region of the 16S rRNA gene, we identified a total of 5,132 OTUs based on 97% similarity during the whole sampling period. The most dominant bacterial phyla were Proteobacteria (31.3%), followed by Actinobacteria (24.8%), Cyanobacteria (10.8%), Bacteroidetes (10.4%), Planctomycetes (8.2%), Verrucomicrobia (5.4%), Chlorobi (2.2%), Gemmatimonadetes (1.9%), Acidobacteria (1.6%), and Chloroflexi (1.2%) at three sites across the whole sampling period at phylum level (Figure 3A). Overall, the variation trends of bacterial community composition at the internal and exit sites were quite similar, which were largely different from bacterial community composition at the inlet site.

For further classification, most of the proteobacterial OTUs classified as Alpha- and Betapeoteobacteria exhibited much higher abundance at the inlet site (16.5% and 16.4%, respectively) than other two sites (11.2% and 9.5% at the internal site, 10.9% and 8.9% at the exit site) (Figure 3B). In addition, the Gammaproteobacteria exhibited higher relative abundance at internal and exit sites (5.9% and 4.8%, respectively) than at the inlet site (3.7%). The relative abundance of both Acidimicrobiia and Actinobacteria, the most dominant actinobacterial OTUs, were relatively stable across the sampling period at the inlet site (with averages of 9.4% and 15%, respectively). In contrast, the fluctuation of Acidimicrobiia abundance was observed both at internal and exit sites (ranging from 4.3% to 12% at the internal site and from 4.4% to 13% at the exit site). However, the relative abundance of Actinobacteria (at the class level) was quite stable at the internal and exit sites (with an average of 15.6% at the internal site and 15% at the exit site). Synechococcophycideae, as the most abundant cyanobacterial OTUs always maintained at lower relative abundance during the sampling period at the inlet site (with an average of 3%), except in July (almost 30.5%). In contrast, the relative abundance of Synechococcophycideae was much higher at the internal and exit sites, especially from July to September (20.6% and 20.1%, respectively). The peaking value was appeared in July at both internal and exit sites (27.6% and 25%, respectively). As the most dominant taxa of the Bacteroidetes phylum, the Flavobacteriia exhibited the highest relative abundance in April at both internal and exit sites (20.4% and 9%, respectively), and maintained at higher relative abundance from April to June at these two sites (10.6% and 5.6%, respectively) compared with inlet site (1.7%). Additionally, the Sphingobacteriia as the second largest group of the Bacteroidetes phylum exhibited relatively higher abundance at internal and exit sites (2% and 2.1%, respectively) than the inlet site (0.9%) during the whole sampling period. In addition, the OPB56 that represented the most dominant Chlorobi OTUs had a higher relative abundance at the inlet site (3.3%) than the internal and exit sites (1.5% and 1.8%, respectively), especially in April and May.

**Figure 4:**
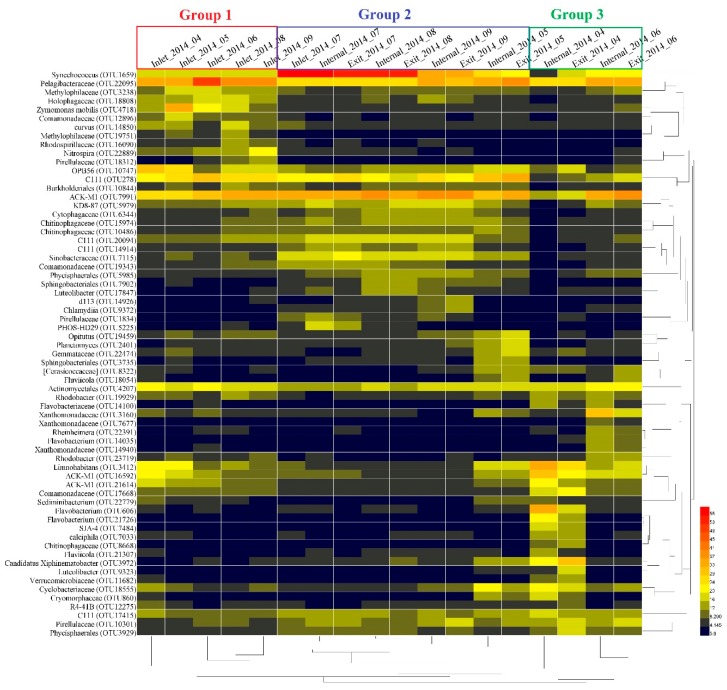
Heat-map analysis for the variations of dominant bacterial OTUs (1% of the total abundance of each sample) based on the Bray-Curtis similarity (OTU level).

The heat-map analysis of the bacterial OTUs with high relative abundance (1% of the total abundance of each sample) revealed that all samples were clustered into three groups (Figure 4). Group 1 was composed of samples from April to September at the inlet site except for July. The second group consisted of samples at both the internal and exit sites in May, August, and September, as well as samples in July at all three sites. Group 3 was mainly composed of samples at both internal and exit sites in April and June. Within these groups, bacterial OTUs including Methylophilaceae (OTU3238 and OTU19751), Holophagaceae (OTU18808), *Zymomonas mobilis* (OTU4718), Comamonadaceae (OTU12896), Rhodospirillaceae (OTU16090), and *Nitrospira* (OTU22889) revealed higher relative abundance in group 1 than other groups. Bacterial OTUs including KD8-87 (OTU5979), Cytophagaceae (OTU6344), Chitinophagaceae (OTU15974 and OTU10486), C111 (OTU20094 and OTU 14914) Sinobacteraceae (OTU7115), Comamonadaceae (OTU19343), Phycisphaerales (OTU5985), Sphingobacteriales (OTU7902), and *Luteolibacter* (OTU17847), Pirellulaceae (OTU1834) and PHOS-HD29 (OTU5225) exhibited higher relative abundance in group 2 than other samples. In addition, the relative abundance of *Opitutus* (OTU19459), *Planctomyces* (OTU2401), Gemmataceae (OTU22474), Sphingobacteriales (OTU3735), [Cerasicoccaceae] (OTU8322), and *Fluviicola* (OTU18054) revealed opposite trends withabundance of *Synechococcus* in group 2. In group 3, bacterial OTUs including Xanthomonadaceae (OTU3160 and OTU14940), *Rheinheimera* (OTU22391), *Flavobacterium* (OTU14035) and *Rhodobacter* (OTU23719) only revealed high abundance in June. In contrast, bacterial OTUs including *Flavobacterium* (OTU606 and OTU21726), SJA-4 (OTU7484), *Calciphila* (OTU7033), Chitinophagaceae (OTU8668), *Fluviicola* (OTU21307), *Luteolibacter* (OTU9323), and Verrucomicrobiaceae (OTU11682) exhibited high abundance only in April in group 3. In addition, *Synechococcus* (OTU1659), Pelagibacteraceae (OTU22095), C111 (OTU278), ACK-M1 (OTU7991), and Actinomycetales (OTU4207) revealed relatively higher abundance than other bacterial OTUs during the sampling period, and bacterial OTUs including *Limnohabitans* (OTU3412), ACK-M1 (OTU16592 and OTU21614) and Comamonadaceae (OTU17668) exhibited higher relative abundance both in group 1 and 3 compared with group 2.

### 2.4. Covariance Analysis of Bacterial Community Composition and Environmental Variables

Marginal test of biotic and abiotic factors in each site based on distance-based linear modelling (DistLM) indicated that the environmental factors exhibited obviously different effects on bacterial community composition between different sampling sites (Table 1). At the inlet site, only DO and temperature significantly affected the variations of bacterial community composition (*p* < 0.05). However, more environmental factors including NH_4_^+^-N, DO, EC, turbidity, temperature, K^+^, Na^+^, Mg^2+^, Cl^−^, and F^−^ exhibited significant effects on bacterial community composition at the internal site. In addition, TN, NH_4_^+^-N, EC, turbidity, temperature, K^+^, Na^+^, Ca^2+^, Mg^2+^, and Cl^−^ significantly affected the bacterial community composition at the exit site. Especially, turbidity extremely significantly affected the composition of the bacterial community at the exit site (*p* < 0.01).

The alpha-diversity indices (including species richness, Pielou’s evenness, and Shannon and Simpson indices) exhibited relatively consistent variation tendencies on a temporal scale and a marked decline appeared at all three sites in July (Table 2). In addition, although the total species were also decreased in July at all sites, the minimum values of total species at the internal and exit sites both appeared in April. On the spatial scale, the values of alpha-diversity indices at the inlet site were obviously higher than internal and exit sites. 

**Figure 5:**
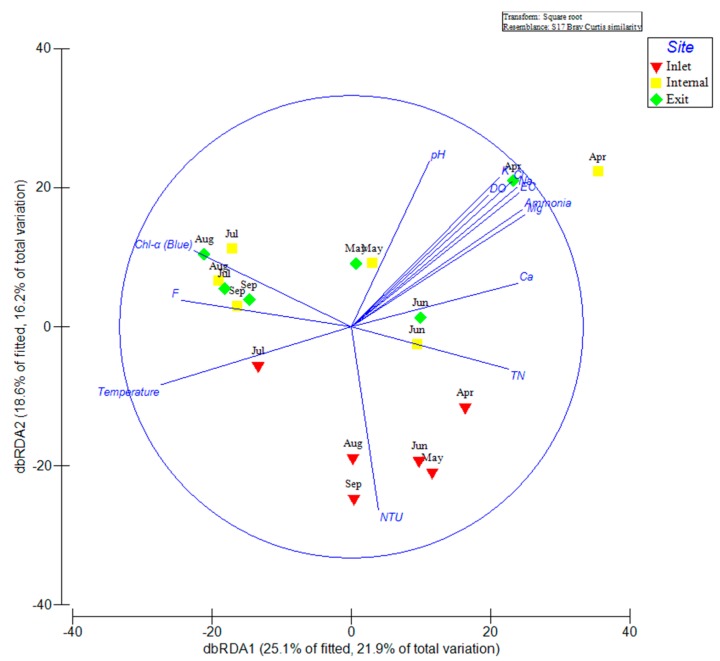
The distance-based linear redundancy analysis (dbRDA) reflecting the distribution of bacterial communities with environmental variables in an estuary reservoir.

The distance-based linear redundancy analysis (dbRDA) visualized the relative contribution of measured environmental variables on total bacterial community composition determined by 16S rRNA gene amplicon sequencing (Figure 5). The distributions of inlet samples were quite different from the samples at other sites. Most inlet samples (except July) clustered loosely and positively correlated with high turbidity and TN. However, samples in July at the inlet site exhibited positive correlation with temperature. In contrast, samples from July to September at both internal and exit sites clustered closely and positively correlated with chlorophyll-α, F^−,^ and high temperature. In addition, samples at internal and exit sites distributed widely in other months and positively correlated with high concentrations of K^+^, Na^+^, Mg^2+^, Cl^−^, NH_4_^+^-N, DO, EC, and pH in April.

### 2.5. Multivariate Analysis of Biotic and Abiotic Factors in the QCS Reservoir

**Figure 6:**
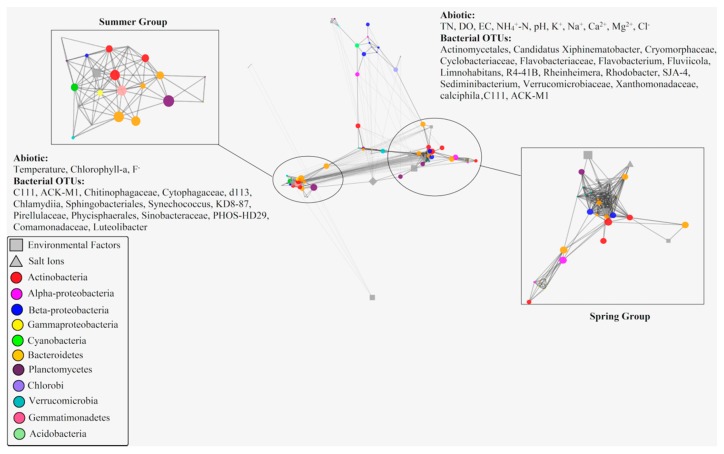
The edge-weighted spring-embedded network based on the Pearson correlation coefficient reflecting the significant correlations between biotic and abiotic factors (node size has reflected the value of betweenness centrality of the variables. Solid lines represent positive correlations and dashed lines represent negative correlation).

A total of 89 measured variables include 65 16S rRNA OTUs contributed >1% to any samples and 24 environmental variables were shown in the single interconnected network. A total of 3916 tested correlations were calculated by using rcor.test in ltm package. During these correlations, only 605 ultimately considered significantly correlated with each other. The significant correlations were further used to construct a visual edge-weighted spring-embedded network, with *r* score as the edge-weight in the network (Figure 6).

By scrutinizing the distribution of biotic and abiotic parameters, it was apparent that the network exhibited a similar distribution trend with the result of dbRDA plot (Figure 5). Most bacterial OTUs and Environmental variables clustered into two obviously different groups (spring group and summer group). Within the edge-weighted spring-embedded network, betweenness is a much more significant indicator of essentiality than other topological parameters. Nodes with high betweenness centrality (large nodes) show high centrality—i.e., higher control over the network. Based on the topological characteristic analysis of nodes within the network, temperature was the only environmental variable with high betweenness centrality (>0.02) in summer group, meanwhile, nine bacterial OTUs including Phycisphaerales (Planctomycetes), Sphingobacteriales (Bacteroidetes), C111 (Actinobacteria), KD8-87 (Gemmatimonadetes), Chitinophagaceae (Bacteroidetes), Cytophagaceae (Bacteroidetes), ACK-M1 (Actinobacteria), and *Synechococcus* (Cyanobacteria) revealed high betweenness centrality (>0.02) within this group. In contrast, environmental variables, including pH, Ca^2+^, NH_4_^+^-N, K^+^, and Cl^−^ with high betweenness centrality (>0.02) during spring group, meanwhile, ten bacterial OTUs including *Flavobacterium* (Bacteroidetes), *Rhodobacter* (Alphaproteobacteria), ACK-M1 (Actinobacteria), *Sediminibacterium* (Betaproteobacteria), Cyclobacteriaceae (Bacteroidetes), Comamonadaceae (Betaproteobacteria), C111 (Actinobacteria), *Limnohabitans* (Betaproteobacteria), and Phycisphaerales (Planctomycetes) exhibited high betweenness centrality (>0.02) in spring groups. Although the biological network indicated these biotic/abiotic factors (bacteria/environmental factors) with high betweenness centrality might play important roles in network composition, there is less evidence to explain how these biotic/abiotic factors affected and controlled the whole network (such as their functions and roles in the ecosystem) due to technical restriction.

**Figure 7:**
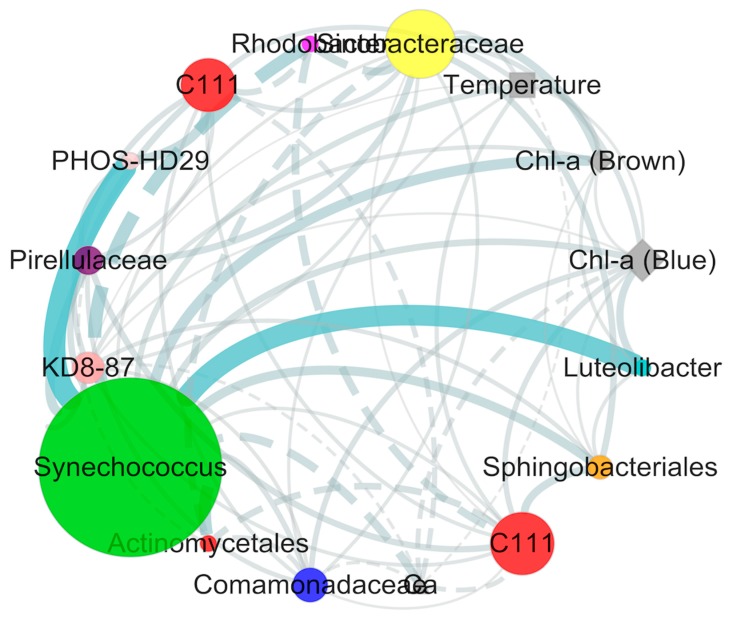
Organic correlation sub-network reflecting the pair-wise correlations between *Synechococcus*, other bacterial OTUsm and environmental factors (node size reflects the value of betweenness centrality of the variables, solid lines represent positive correlations, and dashed lines represent negative correlations).

An organic correlation sub-network was constructed to visualize pair-wise correlations between the dominant *Synechococcus* (OTU1659) and other non-cyanobacterial OTUs (Figure 7). Environmental variables including temperature and Chlorophyll-α were positively associated with *Synechococcus* (OTU1659), and Ca^2+^ as the only salt ions was negatively correlated with *Synechococcus* (OTU1659) in our study. In addition, nine non-cyanobacterial OTUs including Sinobacteraceae (Gammaproteobacteria), C111 (Actinobacteria), KD8-87 (Gemmatimonadetes), Comamonadaceae (Betaproteobacteria), *Luteolibacter* (Verrucomicrobia), Sphingobacteriales (Bacteroidetes), Pirellulaceae (Planctomycetes), and PHOS-HD29 (Proteobacteria) revealedpositive correlations with *Synechococcus* (OTU1659). In contrast, two non-cyanobacterial OTUs including *Rhodobacter* (Alphaproteobacteria) and Actinomycetales (Actinobacteria) negatively correlated with *Synechococcus* (OTU1659).

### 2.6. Shotgun Metagenomic Analysis

Aiming to determine the functional mechanism variations of microbial community during the period of seasonal transition between spring and summer at different sites inside the reservoir, four samples (May–June at the internal site and June–July at the exit site) within this period were selected to assess the variations of enriched set of metabolic genes using the shotgun metagenomic sequencing technology. We obtained about 47.5 Gb of community shotgun metagenomic sequence data in total from four samples inside the reservoir, and a total of 2.76 × 10^2^ million clean reads were generated from the metagenomic dataset of four samples (Table S3). The number of contigs ranged from 153,619 to 219,616 from scaffolds longer than 500 bp when the k-mer value set as 41 across all four samples (Tables S1 and S2). The statistical information including contigs_N50 and N90 length indicated we obtained a relatively high assembly efficiency of the contigs in our study.

Different from the 16S rRNA sequencing technology, the shotgun metagenomic sequencing technology could provide more information to explore the potential functional mechanisms within the microbial community. In this study, the eggNOGs database categories of non-redundant genes indicated that the microbial community inside the reservoir had a relatively high abundance of genes devoted to amino acid transport and metabolism, general function prediction (only), energy production and conversion, replication, recombination and repair, translation, ribosomal structure and biogenesis, cell wall/membrane/envelope biogenesis, inorganic ion transport and metabolism, carbohydrate transport and metabolism and posttranslational modification, protein turnover, chaperones (Gene abundance > 500,000). However, the category of unknown function still accounted for a large proportion of total gene abundance (gene abundance > 1,500,000). Furthermore, thirty COGs with high gene abundance annotated as sulfatase, ABC transporter, DNA polymerase, and other functions were also shown in this Figure.

**Figure 8:**
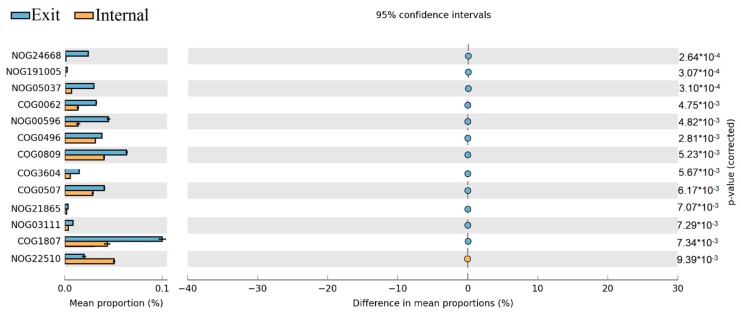
Proportion differences analysis of COG functional categories between internal and exit sites are represented in an extended error plot (the top 80 abundant COG functional categories were selected for analysis). Total mean proportions (%) in the COG categories are exhibited by the bar graph (**left column**); the upper bar graph (blue) represents the samples at the exit site, whereas the other bar graph (yellow) represents the samples at the internal site in each category. The coloured circles corresponding to the (**right column**) (blue and yellow) represent 95% confidence intervals calculated by Welch’s *t*-test. COG functional categories were filtered by *p*-value (0.05) and effect size (0.04).

The Statistical Analysis of Metagenomic Profiles (STAMP) on COGs categories between different sites revealed that NOG24668, NOG05037, COG0062, NOG00596, COG0809, COG0507, and COG1807 have significantly higher abundance at the exit site than at internal site, only NOG22510 were obviously higher at the internal site than at the exit site (Figure 8).

**Figure 9:**
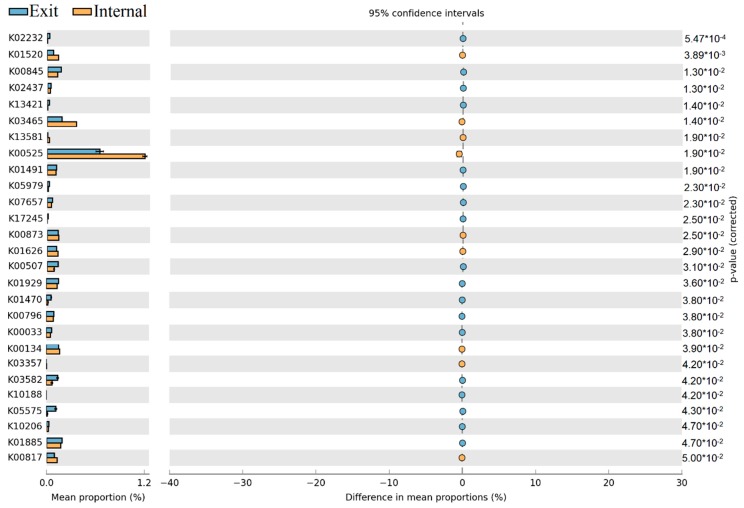
Proportion differences analysis of KEGG functional categories between internal and exit sites are represented in an extended error plot (the top 80 abundant KEGG functional categories were selected for analysis). Total mean proportions (%) in the KEGG categories are exhibited by the bar graph (**left column**); the upper bar graph (blue) represents the samples at the exit site, whereas the other bar graph (yellow) represents the samples at the internal site in each category. The coloured circles corresponding to the (**right column**) (blue and yellow) represent 95% confidence intervals calculated by Welch’s *t*-test. KEGG functional categories were filtered by *p*-value (0.05) and effect size (0.04).

The non-redundant genes also were aligned against the Kyoto Encyclopaedia of Genes and Genomes (KEGG) database using BLAST, to visualize the differences of metabolic pathways within the microbial community. In our study, a total of 4050 KEGG categories were observed within four samples, which are involved in 376 KEGG pathways. Among these KEGG pathways, thirty-two KEGG pathways such as glycolysis/gluconeogenesis, TCA cycle, oxidative phosphorylation, purine/pyrimidine metabolism, carbon metabolism, biosynthesis of amino acids were the most dominant metabolic pathways with an obvious high abundance of all samples. In addition, more than 100 KEGG pathways resulted from STAMP analysis revealed the significant spatial differences between internal and exit sites. We further selected 27 KEGG pathways from these above and displayed in this paper (Figure 9). Among these KEGG pathways, the most notable KEGG pathway was K00525 (ribonucleoside-diphosphate reductase alpha chain), which exhibited much higher relative abundance in all samples, but significantly higher at the internal site.

## 3. Discussion

### 3.1. Temporal and Spatial Dynamics of Microbial Community Composition in the QCS Reservoir

In this study, Illumina MiSeq (16S rRNA sequencing) technology was used to evaluate the microbial community diversity and composition spanning from end spring to summer in different sites of the reservoir. Based on these data, we further used shotgun metagenomic sequencing technology (Illumina HiSeq 4000 platform) to explore functional mechanism variations within the microbial community during the seasonal transition between spring and summer at different sites inside the reservoir.

The variations of microbial community composition indicated microorganisms had similar dominant community structure (at phylum level) at all three sites, but the relative abundance of these dominant bacterial phyla were obvious differences, especially at the inlet site. In addition, the alpha-diversity of bacterial community at the inlet site was also clearly higher than other two sites (Table 2). These changes were mainly due to the differences of water environmental conditions between different sites. At the inlet site, raw water from the Yangtze River runs into the reservoir. This means the aquatic ecological environment at the inlet site was linked with water quality parameters in the Yangtze River characterized for higher concentrations of nutrients (N, P) and turbidity, which is affected by seriously non-point pollution and soil erosion in upstream [23,24,25]. Thus, the inorganic nutrients and organic matter were not restrictive factors for microbial metabolisms at the inlet site. However, the higher turbidity reduced the transparency in a surface water body and further limited the photosynthesis of photosynthetic microorganisms. Therefore, the DO became the restrictive environmental factor. These were coinciding with our experimental data that only DO and temperature were significantly affected the bacterial community composition at the inlet site (*p* < 0.05) (Table 1).

At the inlet site, Methylophilaceae (OTU3238 and OTU19751), *Zymomonas mobilis* (OTU4718), Comamonadaceae (OTU12896), *Limnohabitans curvus* (OTU14850), and Rhodospirillaceae (OTU16090) were representatives of the dominant Alpha- and Betaproteobacterial taxa exhibited higher relative abundance during the sampling period (Figure 4). Strains of family Methylophilaceae have the characteristic function of utilizing methanol/methylamine as the only energy and carbon source, were widely distributed in surface sediment of freshwater lakes [26,27]. Therefore, we assumed that the high abundant Methylophilaceae at the inlet site may be associated with high turbidity, which was derived from soil erosion upstream of the Yangtze River. *Limnohabitans curvus*, as the first described species of the family Limnohabitans, exhibited high relative abundance at the inlet site, which was reported as chemoorganotrophic, aerobic, and facultative anaerobe metabolic types [28]. In addition, these clades were also capable of assimilating glucose and types of small organic acids, excluding amino acids [28]. This implied a potential association between *Limnohabitans curvus* and the high concentration of total organic carbon (TOC) in raw water from the Yangtze River. Another dominant family, Comamonadaceae of Betaproteobacteria, was difficult to obtain more specific information to explain the high abundance at the inlet site, due to a large diversity of phylogeny and functions within this family [29]. The Alphaproteobacteria represented by family Rhodospirillaceae with high abundance at the inlet site have been considered with varying metabolic types, including photoheterotrophs, photoautotrophs, and chemoheterotrophs [30]. In contrast, internal and exit sites were midstream and downstream of the reservoir, respectively. The water flow velocity obviously declined, and have sufficient retention times for purification to increase the transparency of the water column in these areas. The water quality parameters were also indicated that the concentrations of TN, TP and turbidity were remarkably decreased at internal and exit sites than at the inlet site (Figure 1). To some extent, the higher transparency and lower nutrient level at these sites reduced the diversity of the bacterial community and increased the potential possibility of cyanobacterial proliferation in surface water. In addition, there were some potential associations between increased cyanobacterial abundance and reduced diversity of the bacterial community [21,22], which could partly explain the remarkable decline of alpha-diversity indices in July inside the reservoir. In addition, the dominant environmental factors were consistent at both internal and exit site (Table 1), which implied that the microbial community compositions were similar at two sites. The dbRDA plot showed that all July samples grouped together (Figure 5). Combined with the results of the heat-map (Figure 4) and network (Figure 6), we found most of the dominant bacterial taxa in July exhibited positive correlations with temperature. Thus, we speculated that the temperature was the key factor for the composition of the bacterial community in July at all three sites. Additionally, the increased water temperature further promoted some kinds of mesophile bacterial growth. In addition, the bacterial OTUs were strongly connected (negatively correlated) between spring and summer groups within the network (Figure 6). To some extent, this implied that the dynamics and continuity of bacterial community composition varied seasonally inside the reservoir, although some variations in the short-term (days or one week) might be ignored in our study. However, high-frequency sampling in the short-term would perform inside the reservoir, which could further validate these conclusions. While we also found few bacterial OTUs were both excluded from summer and spring groups in the network, these bacterial OTUs exhibited relatively lower abundance inside the reservoir and were less connected with other biotic/abiotic factors, which implied little dependence of these bacterial OTUs on other biotic/abiotic factors.

The *Synechococcus* was the most common Cyanobacteria in coastal areas, which was found to have high abundance in the Yangtze estuary during the summer season in history [9,31]. Early studies have shown that the counterparts of *Synechococcus* in marine ecosystems were found to have the capability to utilize nitrate, ammonia, or urea as nitrogen sources [32], but we are still unclear whether the *Synechococcus* in the estuarine ecosystem has a similar capacity or not. The molecular ecological network further indicated that some bacterial OTUs positively correlated with the increased *Synechococcus*, which implied that co-occurrence correlations probably existed between these bacterial taxa and *Synechococcus* (Figure 7). Among these bacterial OTUs, the C111 of actinobacterial phylum were found to have strong connections with Synechococcaceae in a previous study [33], which indicated that the C111 clades might depend on the carbon source released by these cyanobacterial species. Additionally, some other bacterial OTUs of Betaproteobacteria and Bacteroidetes phyla were also found to have similar functional relationships with the dominant *Synechococcus* (Figure 7). The results were consistent with previous study that these bacterial clades have a similar tendency to increased cyanobacterial abundance, and assimilated dissolved organic matters derived from cyanobacterial cell metabolism as their carbon sources [34]. 

### 3.2. The Variations of Ecological Functions within the Microbial Community during the Period of Later Spring/Early Summer

The results of dbRDA plot demonstrated that the microbial community composition was obviously different between samples in later spring and early summer both at internal and exit site, which implied the potential ecological functions of the dominant microbial community also changed obviously from later spring to early summer at these sites (Figure 5). Subsequently, the functional annotations through eggNOG were shown that the relative abundance of some COG/NOGs taxonomies in critical metabolic reactions was significantly enhanced at the exit site (*p* < 0.05). These COG/NOGs include COG0062, NOG00596, COG0809 and COG1807 (Figure 8). The COG0062 was annotated as ADP-dependent NADHX epimerase, which played important roles in the course of carbohydrate transport and metabolism. NOG00596 was annotated as AMP-binding protein, which played key roles in energy production and conversion. COG0809 and COG1807 were both annotated as glycosyl transferase, which played important roles in translation/ribosomal biogenesis and cell wall/membrane/envelope biogenesis, respectively. These results indicated that the activities of key enzymes involved in carbohydrate transport and metabolism, energy production and conversion, translation/ribosomal biogenesis and cell wall/membrane/envelope biogenesis were significantly enhanced at the exit site. In contrast, only NOG22510 exhibited higher relative abundance at internal site than exit site (Figure 8). The NOG22510 was further annotated as TGFb_propeptide, which was correlated with Beta binding protein and was a main factor controlling cell cycle control/division. Thus, we speculated that the course of cell cycle control/division was more active at internal site than exit site. It was notable that in STAMP analysis, K00525 exhibited significantly higher abundance at internal site than exit site (Figure 9). K00525 was annotated as ribonucleoside-diphosphate reductase, mainly involved in the courses of Purine metabolism (ko00230) and Pyrimidine metabolism (ko00240). These reactions were mainly provided the raw material for DNA synthesis. This result was consistent with the conclusion of COG/NOGs variation analysis that the course of cell cycle control/division was enhanced at an internal site. In addition, it’s important to note that some changes of metabolisms and functions of cells within the bacterial community in short-term (days or one week) might be ignored due to the monthly sampled intervals in our study. Previous study indicated that although the bacterial community composition retained relatively stable over weeks or a month, obvious dynamic of bacterial community composition were observed within short-term (days or one week) [17,35]. This could result in the sharp shifts of potential cell metabolisms within the microbial community during a short time, which would be missing in our study. 

Combined with variations of environmental factors, we can found that the dissolved oxygen and water pH at the internal site was much higher than at the exit site from May to July (Figure 1). We speculated that the respirations of microbial community at the exit site were much stronger than internal site, which could deplete more dissolved oxygen and accumulate more CO_2_ in the water, which resulted in a lower concentration of dissolved oxygen and pH. Besides, the concentration of TP has obviously decreased at the exit site after June compared with the internal site (Figure 1). Phosphorus was an essential nutrient element for the bacterial community in aquatic ecosystems, which played important roles in cell metabolisms and cell structures [36,37]. Early studies indicated that the bacteria have higher cellular requirements for phosphorus relative to carbon in freshwater lakes [38]. Therefore, we speculated that the obviously decreased of TP concentration at the exit site mainly correlated with the increased metabolic activity of carbohydrate transport and metabolism, energy production and conversion, translation/ribosomal biogenesis and cell wall/membrane/envelope biogenesis in this area.

In this study, we used both 16S rRNA sequencing and shotgun metagenomic sequencing technology to detect the diversity and functions of the bacterial community in samples at internal and exit sites during the period of later spring and early summer. The majority bacterial community structure characterized by the shotgun metagenomic sequencing approach was quite similar to the results based on the 16S rRNA sequencing technology. However, there are a few discrepancies in the classification of some individual bacterial taxa by using these two approaches. For example, the relative abundance of *Synechococcus* was obviously higher in June at the exit site by using shotgun metagenomic sequencing technology than the 16S rRNA sequencing technology. These were likely caused by different sequencing procedures between shotgun metagenomic sequencing and 16S rRNA sequencing technology. As such, the 16S rRNA targeted sequencing included extra PCR steps, and other reasons including primer bias or suboptimal PCR conditions in the process [39,40,41]. In addition, the eggNOG database could provide accurate clusters of orthologous groups’ information on proteins, but a great amount of gene was classified as unknown functions. These together indicated that although the shotgun metagenomic sequencing technology could reflect the functional characteristics of microbial community to some extent, there is still a certain gap between the real functions of microorganisms and environments. Furthermore, some specific metabolic pathways were still not clear, which need to be further improved and perfected. 

## 4. Conclusions

To fully understand the dynamics of bacterial community composition during spring and summer in a large estuary reservoir, the 16S rRNA sequencing technology was used to assess characteristics of the bacterial community in different sites monthly. Moreover, the shotgun metagenomic sequencing technology was used to further detect the variations of potential functional mechanisms within the microbial community during the seasonal transition from later spring to early summer. The 16S rRNA sequencing data indicated that obvious differences of bacterial community composition at different sites inside the reservoir. Particle-associated bacterial taxa exhibited obviously higher abundance at the inlet site than at two other sites. In contrast, heterotrophic bacterial taxa exhibited higher abundance with increased *Synechococcus* at internal and exit sites during summer. Correlation analysis indicated temperature was the major factor contributing to the increase of the abundance of *Synechococcus*. The shotgun metagenomic sequencing data indicated that the carbohydrate transport and metabolism, energy production and conversion, translation/ribosomal biogenesis, and cell wall/membrane/envelope biogenesis were significantly enhanced at the exit site. However, the course of cell cycle control/division was more active at the internal site.

## 5. Materials and Methods

### 5.1. Sampling Sites and In Situ Measurements

**Figure 10:**
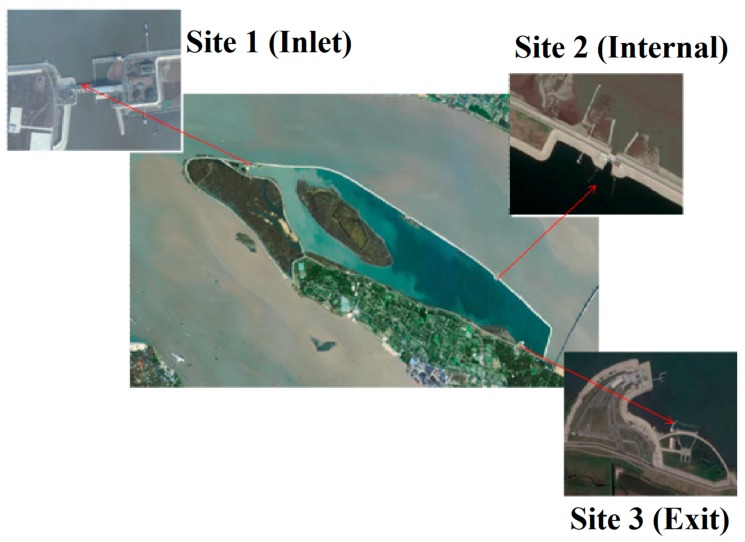
Aerial schematic of the Qingcaosha (QCS) Reservoir and annotated sampling locations (Site 1 (Inlet), Site 2 (Internal), and Site 3 (Exit)).

QCS Reservoir is the largest estuary reservoir in China located at the Yangtze estuary area near Shanghai (Figure 10). The reservoir covers a total catchment area of 66.27 km^2^, with a depth ranged from 2.5 to 13.5 m. Its main purpose is compensating for drinking water shortage in Shanghai, which inputs high turbidity water from the Yangtze River estuary and outputs clean water to water plants after the self-purification in the reservoir [2,8,42]. During our study, we set three sampling sites along the reservoir. The raw water entered the reservoir from the inlet site. The internal and exit sites represented the midstream and downstream of the reservoir, respectively (Figure 10). All water samples were collected at a depth of 0.5 m below the surface monthly from April to September 2014, which is the warm seasons from spring to summer with a high risk of cyanobacterial bloom [9,31]. Water temperature and dissolved oxygen (DO) were detected in situ using multi-parameter water quality analyser (Multi3410, WTW Company, Weilheim, Germany).

### 5.2. Physic-Chemical Parameters and Environmental Factors

Physico-chemical parameters and environmental factors include pH, electrical conductivity (EC), turbidity, total phosphorus (TP), and ammonium nitrogen (NH_4_^+^-N) were analysed according to water and wastewater monitoring analysis standard methods. Total carbon (TC), total nitrogen (TN), total organic carbon (TOC), and inorganic carbon (IC) were detected by using a Multi N/C 3100 Analyser (Jena, Germany). The concentrations of chlorophyll-α (Chl-α), which represented phytoplankton biomass, were measured using PHYTO-PAM phytoplankton analyser (Waltz, Germany) [43]. The PHYTO-PAM phytoplankton analyser could distinguish different types of phytoplankton, like chlorophyta, diatoms, and cyanobacteria, based on the specific fluorescence excitation properties of differently pigmented phytoplankton groups and exhibited high detection precision. After all water samples were filtrated through 0.45 μm Cellulose Acetate filter membranes, the concentrations of K^+^, Na^+^, Ca^2+^, Mg^2+^, Al^3+^, and Si^4+^ ions were detected by inductively-coupled plasma (ICP) spectroscopy. The F^−^, Cl^−^, and SO_4_^2−^ ions contents were detected using a Metrohm 830 ion chromatographer [8].

### 5.3. DNA Extraction

A total of 500 mL volume water samples at each site were filtrated through 0.22 μm cellulose acetate filter membranes immediately on receipt at Shanghai Jiaotong University (SJTU, Shanghai, China). Total DNA was extracted directly from the same amount of membranes using an E.Z.N.A. Water DNA Kit (Omega, Irving, TX, USA) in according to the manufacturer’s specifications. To ensure the DNA samples were adequate for metagenomic analysis, we conducted six replicates for DNA extraction per water sample using the same DNA extraction approach described previously.

### 5.4. The 16S rRNA Gene Sequencing via PCR Amplification

To determine the diversity and variation of bacterial community composition in different sites of the reservoir, we used PCR amplification for each water sample with the 515F/806R primer set which could amplify the V4 region of the 16S rRNA gene. This primer set exhibited lower biases and more accurate taxonomic and phylogenetic information for individual bacterial taxa [44]. The PCR amplifications were performed in 25 μL reaction mixtures containing 5.0 μL 5* Q5 Reaction Buffer, 5.0 μL 5*Q5 GC high Enhancer, 2.5 mM of dNTPs 2.0 μL, 1.0 μL of forward and reverse primers (10 μM each), 0.25 μL of Q5 DNA Polymerase (5 U/μL), and 1 μL DNA template (20 ng/μL each). The following PCR cycling processes included an initial denaturation at 98 °C for 5 min, then followed by 27 cycles of denaturation at 98 °C for 30 s, annealing at 50 °C for 30 s, extension at 72 °C for 30 s, and a final elongation at 72 °C for 5 min. The 16S rRNA PCR products were then further purified using MinElute PCR Purification Kit (Qiagen, Gmbh, Germany).

After purification, paired-end amplicon sequencing (2 × 150 bp) were sent to Personal Biotechnology Co., Ltd. (Shanghai, China) for Illumina sequencing. Raw data were processed according to procedures described previously [45,46], using the Quantitative Insights into Microbial Ecology (QIIME) pipeline (version 1.7.0, http://qiime.org/) for quality control. Uchime was implemented in Mothur (version 1.31.2, http://www.mothur.org/) to identify and remove chimeric sequences [47,48]. At this stage, sequences less than 150 bp in length, which means quality less than 20, and sequences containing Ns and any ambiguous bases pairs were eliminated from pair-end sequence reads. Sequences were subsampled at a level of 21,869 reads for each sample before the further analysis. Purified sequences were binned into operational taxonomic units (OTUs) based on a 97% identity threshold, while the longest sequence of each OTU was selected as the representative sequence for that OTU based on UCLUST algorithm using QIIME [49]. The taxonomic identity of OTUs was aligned and compared with Ribosomal Database Project classifier (Release 11.1, http://rdp.cme.msu.edu/), SILVA database (Release 119, http://www.arb-silva.de), and the Greengenes database (97% taxonomy) (Release 13.8, http://greengenes.secondgenome.com/), which were used for taxonomy assignment of bacteria and archaea [50,51,52]. The raw sequencing datasets were available from NCBI Sequence Read Achieve under BioProject PRJNA397386.

### 5.5. Statistical Analysis of the 16S rRNA Sequencing Data

To better understand the dynamic distributions of dominant bacterial OTUs, we selected the bacterial OTUs which were detected at least 20% samples and contributed to at least 1% of the total abundance of each sample. The relative abundance of these OTUs was further transformed by square root to reduce the disturbance of highly abundant OTUs in the analysis progress. A heat-map was constructed for cluster analysis of the distributions of these bacterial OTUs based on Bray-Curtis similarity at the genus level.

Distance-based linear models (DistLM) were created to model and evaluate the contribution of each measured environmental variable on variations of microbial community composition by using PRIMER v6 and PERMANOVA+ (PRIMER-E Ltd., Plymouth, UK). Alpha-diversity parameters including total species, species richness, Pielou’s evenness, Shannon and Simpson indices between samples were calculated using PRIMER v6. Furthermore, Distance-based redundancy analysis (dbRDA) was implemented to assess the correlations between environmental factors and distributions of microbial community in spatial and temporal scales. The degree of paired correlations between each biotic and abiotic factors across the whole sampling period was calculated using Pearson’s correlation coefficient (*r*). Highly abundant OTUs, which were observed at least four samples (>20% samples) and contributed at least 1% to any given samples were selected. All original abundance values of these OTUs were retained without any alteration. Both Pearson’s correlation coefficient (*r*) and *p*-value were calculated pairwise based on a rcor.test algorithm by using ltm package in R (version 3.2.0) for each OTU. During the operational processes, the *p*-value was generated with each counterpart correlation and the false discovery rate was constantly kept below 5% based on the Benjamini-Hochberg procedure [53]. Based on these significant correlations, a visualized edge-weighted spring-embedded network was generated by using Cytoscape package (version 3.2.1), which was according to *r*-value as the edge-weighted of the network. Within the network, relevant topological and node/edge metrics including betweenness centrality was also enumerated through the network analysis plug-in [54].

### 5.6. Shotgun Metagenomic Analyses

Shotgun metagenomic sequencing was used for the same DNA extracts of four selected samples (May–June at the internal site and June–July at the exit site) inside the reservoir. The genome DNA was mechanically sheared into ~300 bp fragments using an M220 Focused-ultrasonicator^TM^ (Covaris Inc., Woburn, MA, USA). Meanwhile, paired-end library (2 × 150 bp) was constructed. After the procedures of DNA templates enrichment and bridge PCR amplification, the paired-end reads (2 × 150 bp) were sequenced by Illumina HiSeq 4000 at Majorbio Bio-Pharm Technology Co., Ltd. (Shanghai, China) using Truseq SBS Kit v3-HS following the standard protocol (www.illumina.com). All the raw metagenomic datasets have been submitted into NCBI Sequence Read Achieve under accession BioProject PRJNA393607.

### 5.7. Sequence Quality Control and Assembly

In order to improve the quality and reliability of subsequent analysis, Seqprep (https://github.com/jstjohn/SeqPrep) software was used for quality control. Sickle (https://github.com/najoshi/sickle) was used to remove reads of which the length is less than 50 bp, mean Quality is less than 20 and contain N [55]. The clean reads were assembled using SOAPdenovo (Version 1.06, http://soap.genomics.org.cn/) based on De-Brujin graph with a range of k-mers (39–47). The length of scaffolds over than 500 bp was chosen for further analysis. Based on the quality and quantity of the scaffolds assembly, the maximum number of the scaffold and the peak value of N50 and N90 were obtained when the k-mer value was set at 41. New contigs were extracted when the scaffolds were broken from gaps inside. Then, the contigs with length over 500 bp were further used for prediction and annotation. The statistics of assembly results can be found in Table S1.

### 5.8. Gene Prediction, Taxonomy, and Functional Annotation

Open reading frames (ORFs) (Table S2) of contigs in each sample were predicted using the MetaGene software (http://metagene.cb.k.u-tokyo.ac.jp/). The ORFs with length over 500 bp were extracted and translated to amino acid sequences. In order to better understand the commonness and difference between samples, the dynamic changes of abundance of microorganisms (or genes) were compared. Moreover, the non-redundant gene catalogue was constructed using CD-HIT software (http://www.bioinformatics.org/cd-hit/) (Parameters: 95% identity, 90% coverage), then the longest genes of each cluster were chosen as representative sequences. High-quality reads were aligned to the Non-redundant gene catalogue (95% identity) using SOAPaligner software (http://soap.genomics.org.cn/) and the abundance of each Non-redundant genes was counted for each sample. Non-redundant gene catalogue was aligned against eggNOG database (cut-off: *e* value < 1 × 10^−5^) by BLASTP (BLAST Version 2.2.28+, http://blast.ncbi.nlm.nih.gov/Blast.cgi) for Clusters of orthologous groups (COGs) of proteins assignment and the Kyoto Encyclopedia of Genes and Genome (KEGG) database (cut-off: *e* value < 1 × 10^−5^) by BLAST (BLAST Version 2.2.28+, http://blast.ncbi.nlm.nih.gov/Blast.cgi). The catalogue was also assigned KEGG functional annotation by KOBAS 2.0 (KEGG Orthology Based Annotation System, http://kobas.cbi.pku.edu.cn/home.do). The pairwise statistical comparative analyses of COG and KEGG functional classification between samples were realized by STAMP software (http://kiwi.cs.dal.ca/Software/STAMP). The significance of the results was evaluated based on the Welch’s-test. The COGs and KEGG categories that were larger than 1% of total abundance in each sample were selected and then calculated using PRIMER v6 and PERMANOVA+ (PRIMER-E Ltd., Plymouth, UK). Further, the independent sample *t*-test was calculated to compare the values of these COGs and KEGG categories between site 2 and 3 using SPSS software (SPSS v22. Inc., Armonk, NY, USA).

## Figures and Tables

**Table 1:** DistLM results of abundant bacterial community data against environmental variables (999 permutations).

Variables	Inlet		Internal		Exit	
	Pseudo-F	*p*	Pseudo-F	*p*	Pseudo-F	*p*
TC	0.751	0.933	0.731	0.655	1.528	0.059
TOC	0.734	0.943	0.673	0.828	0.673	0.971
IC	0.717	0.952	0.718	0.716	0.967	0.391
TN	1.352	0.059	1.669	0.090	1.923	**0.020**
TP	1.231	0.172	0.883	0.470	1.215	0.247
NH_4_^+^-N	1.368	0.052	2.437	**0.045**	1.658	**0.034**
pH	1.205	0.165	1.839	0.057	1.291	0.148
DO	1.426	**0.023**	2.119	**0.040**	1.262	0.193
EC	1.158	0.239	2.628	**0.029**	1.802	**0.021**
Turbidity	0.813	0.802	2.366	**0.018**	2.094	**0.003**
Temperature	1.426	**0.029**	2.417	**0.030**	1.738	**0.033**
Cl^−^	1.164	0.199	2.674	**0.018**	1.797	**0.008**
SO_4_^2-^	0.730	0.917	0.986	0.381	1.209	0.205
F^−^	1.337	0.089	2.357	**0.011**	1.441	0.134
Ca^2+^	0.733	0.950	1.637	0.097	1.807	**0.033**
Mg^2+^	0.970	0.528	2.524	**0.024**	1.849	**0.015**
Na^+^	1.216	0.149	2.686	**0.019**	1.803	**0.010**
Al^3+^	0.910	0.642	1.234	0.191	1.516	0.080
K^+^	0.962	0.540	2.438	**0.023**	1.889	**0.012**
Si^4+^	0.683	0.978	0.795	0.605	0.863	0.604
Chl-α	1.345	0.167	1.348	0.170	1.172	0.252

**Bold**: Significantly correlated with community structure at *p* < 0.05.

**Table 2:** The diversity of microbial community composition between samples.

Sample	Total Species	Species Richness	Pielou’s Evenness	Shannon	Simpson
Inlet_2014_04	1380	144.6	0.6997	5.059	0.9762
Inlet_2014_05	1349	141.4	0.6724	4.846	0.9708
Inlet_2014_06	1601	167.8	0.6644	4.903	0.9536
Inlet_2014_07	1131	118.5	0.6019	4.232	0.9084
Inlet_2014_08	1382	144.8	0.6969	5.039	0.9739
Inlet_2014_09	1744	182.8	0.6991	5.218	0.9718
Internal_2014_04	701	73.41	0.6637	4.349	0.9685
Internal_2014_05	1138	119.2	0.6798	4.784	0.9724
Internal_2014_06	1127	118.1	0.6946	4.881	0.9747
Internal_2014_07	1015	106.3	0.5991	4.148	0.9185
Internal_2014_08	1107	116	0.6271	4.395	0.9375
Internal_2014_09	1163	121.9	0.6591	4.652	0.9627
Exit_2014_04	990	103.7	0.7044	4.859	0.9806
Exit_2014_05	1128	118.2	0.6642	4.667	0.9661
Exit_2014_06	1251	131.1	0.7031	5.014	0.9743
Exit_2014_07	1133	118.7	0.6150	4.325	0.9294
Exit_2014_08	1173	122.9	0.6311	4.459	0.9361
Exit_2014_09	1265	132.6	0.6752	4.823	0.9672

## Supplementary Materials

The following are available online at http://www.mdpi.com/2072-6651/10/8/315/s1, Table S1: Statistics of assembly result, Table S2: Statistics of predicted ORF, Table S3: Statistics of clean data.
