# Supplementary material for: Variations of Bacterial Community Composition and Functions in an Estuary Reservoir during Spring and Summer Alternation
Source: Toxins (Basel). 2018 Aug 6;10(8):315. doi: 10.3390/toxins10080315 (PMC6116017; doi:10.3390/toxins10080315)
Supplement: Supplementary file 1 [file toxins-10-00315-s001.pdf]

Supplementary Materials: Variations of Bacterial Community Composition and Functions in an Estuary Reservoir during Spring and Summer Alternation

Zheng Xu, Shu Harn Te, Cong Xu, Yiliang He and Karina Yew-Hoong Gin

Table S1. Statistics of assembly result.

Sample	Kmer	Contigs	Contigs bases (bp)	N50 (bp)	N90 (bp)	Max (bp)	Min (bp)
2014-05 (Internal)	K41	157,940	170,913,569	1,167	572	49,563	500
2014-06 (Internal)	K41	153,619	184,578,271	1,366	592	151,342	500
2014-06 (Exit)	K41	219,616	259,171,705	1,321	589	138,130	500
2014-07 (Exit)	K41	158,160	193,131,450	1,393	591	78,191	500

Table S2. Statistics of predicted ORF.

Sample	ORFs	Total length (bp)	Average length (bp)	Max (bp)	Min (bp)
2014-05 (Internal)	260,724	142,152,317	545	18,716	500
2014-06 (Internal)	273,994	152,791,055	558	13,839	500
2014-06 (Exit)	380,390	222,162,183	584	27,486	500
2014-07 (Exit)	294,093	171,556,187	583	33,918	500

Table S3. Statistics of clean data.

Sample	Clean reads	Clean bases (bp)	Percent in raw reads (%)	Percent in raw bases (%)
2014-05 (Internal)	71,613,537	10,415,994,537	95.73	92.82
2014-06 (Internal)	62,711,213	9,109,524,058	95.28	92.27
2014-06 (Exit)	73,004,426	10,620,530,364	95.63	92.74
2014-07 (Exit)	68,901,847	9,989,982,838	95.06	91.89
